# Photon-Counting CT in Oral and Maxillofacial Imaging: Clinical Applications, Dose Efficiency, and Future Perspectives

**DOI:** 10.3390/diagnostics16213617

**Published:** 2026-11-02

**Authors:** Atanaz Darvizeh, Fatemeh Darvizeh, Luigi Calegari, Arosh S. Perera Molligoda Arachchige, Cinzia Maspero

**Affiliations:** 1 Fondazione IRCCS Cà Granda Ospedale Maggiore Policlinico, 20122 Milan, Italy; atanaz.darvizeh@unimi.it (A.D.); cinzia.maspero@unimi.it (C.M.); 2 Department of Biomedical, Surgical and Dental Sciences, School of Dentistry, University of Milan, 20122 Milan, Italy; 3 Department of Diagnostic Imaging and Stereotactic Radiosurgery, CDI Centro Diagnostico Italiano S.p.A., Via Simone Saint Bon 20, 20147 Milan, Italy; 4 4C-Studio Polispecialistico, Via Vittor Pisani 14, 20124 Milan, Italy; luigi.calegari@studiopoli4c.com; 5 Postgraduate School of Diagnostic Radiology, University of Milan, Via Festa del Perdono 7, 20122 Milan, Italy

**Keywords:** photon-counting detector CT, oral and maxillofacial radiology, cone-beam CT, dental imaging, metal artifact reduction, spectral imaging, radiation dose

## Abstract

Photon-counting computed tomography (PCCT) is an emerging imaging technology that combines ultra-high spatial resolution, improved dose efficiency, reduced electronic noise, and intrinsic spectral imaging. These characteristics are particularly relevant to oral and maxillofacial radiology, where accurate depiction of small osseous and dental structures is often required in the presence of metallic restorations or implants. This review summarizes the technical principles of PCCT and examines its current and potential clinical applications across dental and maxillofacial imaging. Available evidence indicates that PCCT can improve visualization of fine root canal anatomy, periodontal and trabecular bone structures, peri-implant interfaces, subtle maxillofacial fractures, and complex sinonasal anatomy. Its spectral capabilities, including virtual monoenergetic imaging and material decomposition, may substantially reduce metal-related artifacts and improve tissue characterization. Early studies also suggest that PCCT can provide high diagnostic image quality at reduced radiation dose, with potential advantages in pediatric imaging and in patients requiring repeated examinations. Beyond morphology, PCCT may support quantitative applications such as bone mineral density assessment, iodine mapping, treatment planning, and the development of imaging biomarkers and artificial intelligence-based analysis. Despite these promising findings, cone-beam CT remains the preferred modality for many routine dental indications because of its accessibility, lower cost, and established clinical workflow. Current evidence for PCCT in oral and maxillofacial radiology is still limited and is largely derived from technical, experimental, and early clinical studies. Further prospective comparisons with cone-beam CT and conventional CT are needed to define task-specific diagnostic benefits, optimized protocols, radiation-dose advantages, and cost-effectiveness. PCCT is therefore best regarded as a promising complementary modality with particular value in diagnostically challenging cases that require very high spatial resolution, spectral information, or effective metal-artifact reduction. Nevertheless, most oral and maxillofacial evidence remains exploratory; improved image-quality metrics should not be equated with superior patient-level diagnostic accuracy. Direct comparisons with optimized CBCT yield heterogeneous, protocol-dependent dose and diagnostic findings. No standardized indication-specific PCCT acquisition framework currently exists for routine oral and maxillofacial imaging. Clinical implementation should therefore be conditional on demonstrable diagnostic benefit at a justified radiation dose and acceptable resource cost.

## 1. Introduction

Oral and maxillofacial radiology (OMFR) is central to the diagnosis, treatment planning, and follow-up of disorders affecting the teeth, jaws, temporomandibular joints, salivary glands, and surrounding craniofacial structures. Cross-sectional imaging is increasingly used in implant planning, endodontics, orthodontics, maxillofacial trauma, odontogenic lesions, oncologic assessment, and postoperative monitoring. These applications require detailed visualization of complex osseous anatomy while minimizing radiation exposure, particularly in younger patients and those undergoing repeated examinations.

Cone-beam computed tomography (CBCT) and multidetector computed tomography (MDCT) are the principal cross-sectional imaging modalities used in OMFR. CBCT provides high spatial resolution at relatively low radiation doses and is well suited to dental and dentoalveolar imaging. However, it has limited soft-tissue contrast and is susceptible to beam-hardening and metal artifacts. MDCT offers superior soft-tissue assessment and is frequently used for trauma, malignancy, and extensive inflammatory disease, but generally provides lower spatial resolution for fine dental structures and may involve higher radiation exposure than CBCT. Both techniques may be substantially affected by artifacts from dental restorations, implants, and orthodontic appliances [1].

Photon-counting detector CT (PCCT) is a next-generation CT technology that directly converts individual X-ray photons into electrical signals while recording their energy levels [2]. Compared with conventional energy-integrating detector CT, PCCT offers higher spatial resolution, improved contrast-to-noise ratio, greater dose efficiency, and intrinsic spectral imaging. In OMFR, these characteristics may improve visualization of fine dental and osseous structures, reduce metal artifacts, and enable material-specific and quantitative imaging applications [1].

This review summarizes the technical principles of PCCT and critically evaluates its current and emerging applications in oral and maxillofacial radiology, with particular emphasis on image quality, metal artifact reduction, radiation dose, quantitative imaging, and future clinical developments. Building upon the earlier review by Zanon et al. [1], the present work incorporates more recent experimental and clinical evidence and extends the discussion beyond conventional dental applications to maxillofacial trauma, sinonasal disease, head and neck oncology, pediatric imaging, and emerging quantitative techniques, while critically examining the methodological limitations and clinical applicability of the available findings.

The specific contribution of this review is an indication-based appraisal of the evidence-to-implementation gap: rather than treating spatial resolution, artifact suppression, or dose efficiency as clinical endpoints in themselves, we examine whether these metrics have been linked to validated diagnostic decisions, how they compare with optimized CBCT, and what protocol and outcome evidence is still required. This is a critical synthesis of published studies, not a formal systematic comparison or quantitative estimate of comparative effectiveness.

### Review Methodology

This narrative review was developed through a structured literature search of PubMed/MEDLINE, Scopus, and Web of Science, covering publications from database inception to August 2026. Search terms included combinations of “photon-counting CT”, “photon-counting detector CT”, “PCCT”, “PCD-CT”, “dental imaging”, “oral and maxillofacial radiology”, “cone-beam CT”, “endodontics”, “periodontal imaging”, “dental implants”, “maxillofacial trauma”, “temporomandibular joint”, “paranasal sinuses”, and “head and neck imaging”. Eligible publications included original clinical investigations, experimental and phantom studies, ex vivo and cadaveric studies, and technically relevant feasibility studies addressing the diagnostic performance, image quality, radiation dose, or potential clinical applications of PCCT in oral and maxillofacial imaging. Relevant reviews were also considered for background information, and their reference lists were screened to identify additional primary studies. Publications lacking direct relevance to the review objectives or sufficient methodological information were excluded. Studies were selected according to their clinical relevance and methodological contribution, with priority given to original investigations and direct comparisons with cone-beam CT or conventional CT.

Findings were synthesized narratively according to clinical application, with particular attention to study design, sample size, technical performance, and clinical applicability. Given the narrative nature of this review, no formal risk-of-bias assessment or quantitative meta-analysis was performed. For interpretation, we distinguish technical or phantom measurements, ex vivo/cadaveric studies, animal investigations, and clinical patient data. These designs differ in their ability to estimate diagnostic accuracy, patient benefit, and real-world dose. Because study selection was narrative and no formal risk-of-bias assessment was undertaken, comparisons should be interpreted as descriptive rather than pooled comparative-effectiveness conclusions.

## 2. Technical Principles of Photon-Counting Detector CT

Conventional CT systems use energy-integrating detectors, in which X-ray photons are converted into visible light by scintillators and subsequently into electrical signals by photodiodes. These detectors measure the total deposited energy rather than individual photons, and the indirect conversion process is associated with electronic noise, loss of spectral information, and limitations in spatial resolution [2,3].

Photon-counting detector CT (PCCT) instead uses semiconductor materials, such as cadmium telluride or cadmium zinc telluride, to directly convert incident X-ray photons into electrical signals. Each photon is individually counted and assigned to a predefined energy bin according to its measured energy. This enables spectral information to be acquired during every scan without requiring a dedicated dual-energy protocol and supports applications such as virtual monoenergetic imaging, material decomposition, quantitative imaging, and improved tissue characterization [3].

The exclusion of low-energy electronic noise and the more efficient use of detected photons improve signal-to-noise and contrast-to-noise ratios, potentially allowing lower-dose acquisitions. In addition, the absence of scintillators and reflective septa permits smaller detector elements and higher spatial resolution, with voxel sizes approaching 100 μm in selected acquisition modes [1]; however, voxel size should not be interpreted as equivalent to true spatial resolution.

The intrinsic spectral capability of PCCT may also facilitate differentiation among enamel, dentin, cortical and cancellous bone, soft tissues, and metallic materials. High-keV virtual monoenergetic reconstructions can reduce beam-hardening and metal artifacts from dental implants and restorations, thereby improving visualization of adjacent anatomical structures [1].

**Figure 1:**
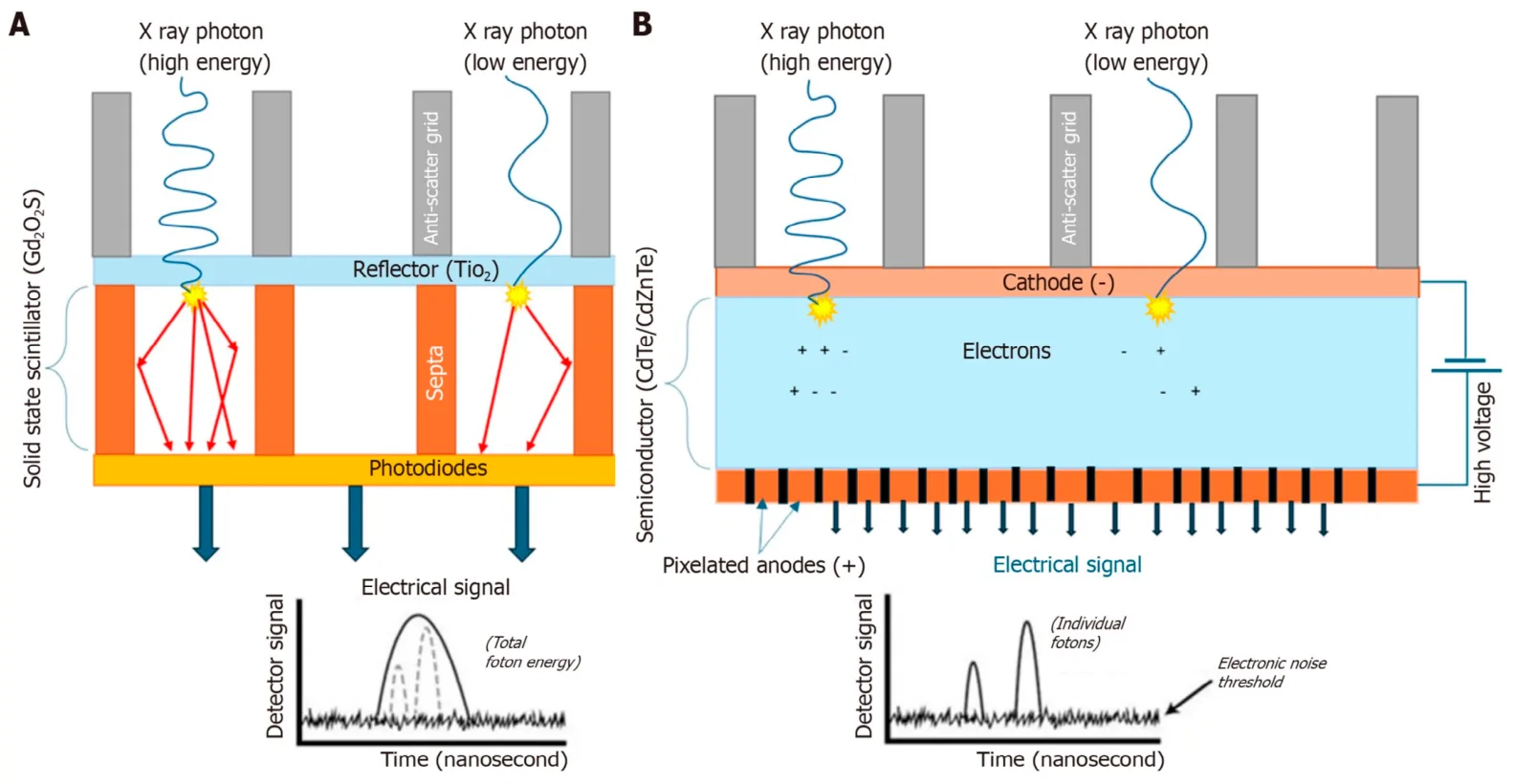
A head-to-head comparison between the energy-integrating (**A**) versus photon-counting detector systems (**B**). Reproduced from Ref. [2].

Compared with conventional multidetector CT, PCCT therefore offers higher spatial resolution, lower electronic noise, improved dose efficiency, routine spectral imaging, and more effective material decomposition. However, current limitations include higher system costs, larger data volumes, greater computational requirements, and detector-related effects such as charge sharing and pulse pile-up, although ongoing technological developments are progressively reducing their impact (Figure 1) [2,3].

## 3. Clinical Applications and Dose Efficiency in Oral and Maxillofacial Imaging

### 3.1. Dental and Alveolar Bone Imaging

**Figure 2:**
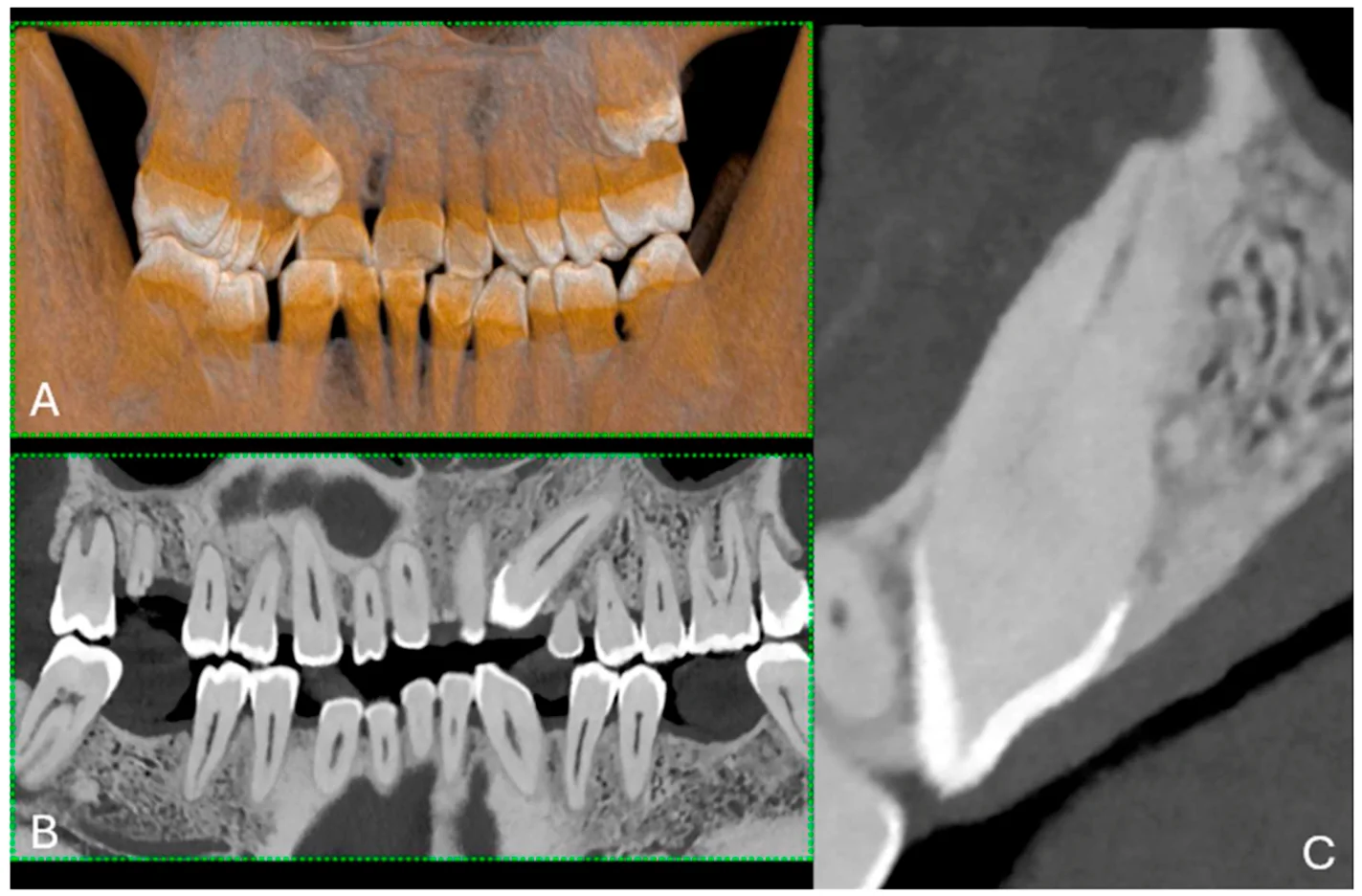
High-resolution dental imaging with photon-counting CT. (**A**) Three-dimensional cinematic rendering of the dentition; (**B**) longitudinal multiplanar reconstruction of the upper and lower jaws; and (**C**) detailed cross-sectional visualization of a single tooth, its root, and the surrounding alveolar bone. The images demonstrate high-resolution dental imaging achievable with clinical photon-counting CT, with a displayed in-plane resolution of approximately 100 μm. Reproduced from Ref. [1].

Dental and alveolar bone imaging represents one of the most promising applications of PCCT in oral and maxillofacial radiology. The combination of ultra-high spatial resolution, improved dose efficiency, and reduced image noise enables detailed visualization of teeth, root morphology, periodontal structures, and alveolar bone. High-resolution multiplanar and three-dimensional reconstructions may therefore provide a comprehensive assessment of dentoalveolar anatomy and serve as a basis for more specific applications in endodontics, periodontal imaging, and implant planning (Figure 2) [1].

#### 3.1.1. Visualization of Fine Endodontic Structures

**Figure 3:**
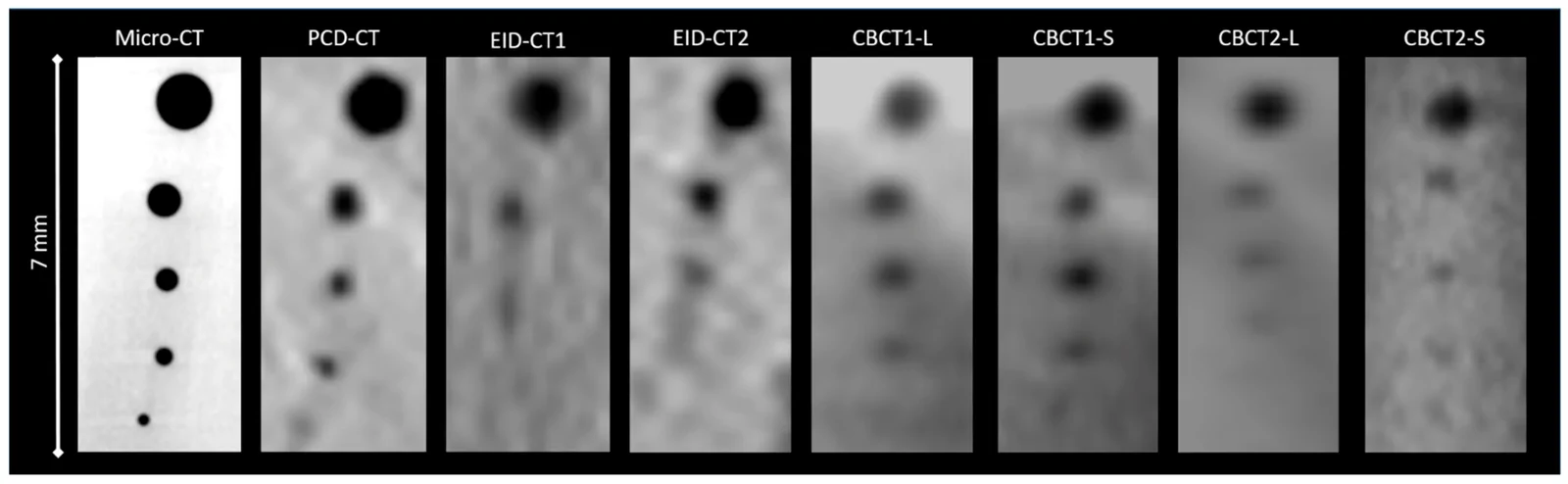
Comparison of artificial accessory root canals across photon-counting detector CT (PCD-CT), energy-integrating detector CT (EID-CT), and cone-beam CT (CBCT) systems, demonstrating the improved visualization of small-caliber endodontic structures with PCCT. Reproduced from Ref. [4].

Rau et al. [4] evaluated the visualization of artificial accessory root canals using PCCT, EID-CT, and CBCT. PCCT demonstrated improved delineation of small-caliber endodontic structures, with reliable visualization of artificial canals as small as 200 μm, whereas conventional CBCT showed reduced reliability for structures below 300 μm (Figure 3). These findings demonstrate the potential of PCCT for high-resolution visualization of fine endodontic anatomy; however, this study did not evaluate dental caries or early enamel demineralization, and its findings should not be extrapolated to caries detection without direct supporting evidence.

**Figure 4:**
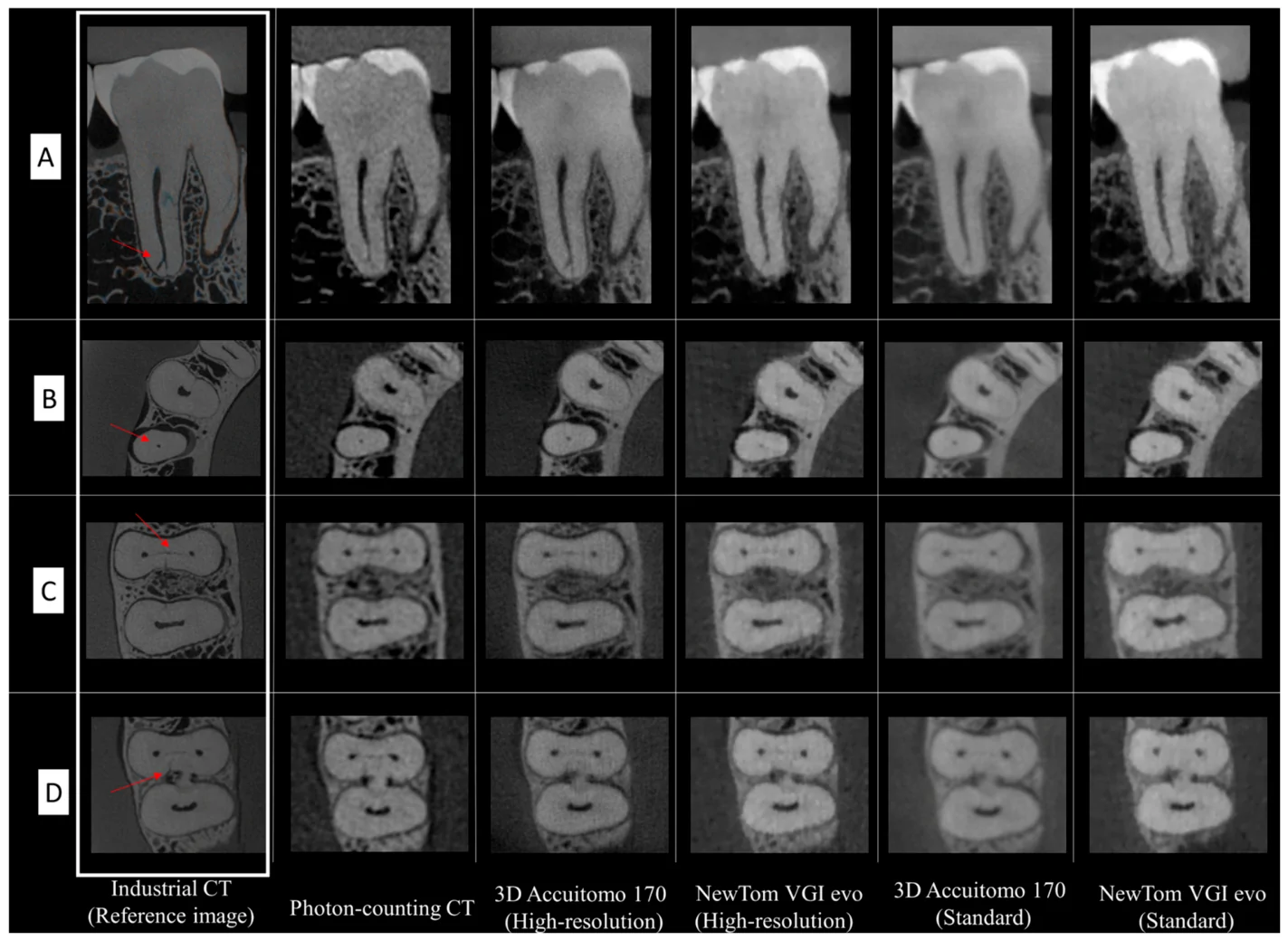
Representative comparison of imaging modalities for challenging endodontic diagnostic tasks, as indicated by the red arrows: (**A**) apical delta, (**B**) narrow root canal, (**C**) isthmus, and (**D**) root crack. Photon-counting CT (PCCT) enables detailed visualization of fine endodontic structures. For root crack visualization, high-resolution (HR) 3D Accuitomo 170 demonstrated significantly better performance than PCCT and NewTom VGi evo, regardless of the acquisition protocol (*p* < 0.05). Root crack visualization was rated as appropriate with HR 3D Accuitomo 170 and acceptable with PCCT, whereas standard-resolution (SR) 3D Accuitomo 170 and both SR and HR NewTom VGi evo protocols provided inappropriate visualization. Reproduced from Ref. [5].

Fontenele et al. [5] compared PCCT with standard- and high-resolution CBCT for challenging endodontic diagnostic tasks, including the assessment of apical delta, narrow root canals, isthmuses, and root cracks (Figure 4). PCCT provided high-quality visualization of fine endodontic structures; however, high-resolution CBCT also demonstrated favorable performance for several tasks. Importantly, the estimated effective dose of the PCCT protocol (0.85 mSv) was higher than that of both standard-resolution CBCT (0.03–0.13 mSv) and high-resolution CBCT (0.01–0.04 mSv), emphasizing the need to consider radiation exposure alongside diagnostic performance [5].

Al-Haj Husain et al. [6] compared PCCT and CBCT in an ex vivo study involving 16 extracted teeth and eight simulated endodontic tasks. At approximately matched effective doses (102, 75, and 13.1 µSv for PCCT versus 108, 78.4, and 14.7 µSv for CBCT at high, standard, and low dose levels, respectively), PCCT demonstrated superior image quality, particularly at high and standard dose levels. Diagnostic accuracy ranged from 80–88% for PCCT and 75–88% for CBCT, although further clinical validation is required [6].

The contrast between these reports is clinically important: the protocol in Fontenele et al. [5] incurred substantially greater effective dose than the evaluated CBCT protocols, whereas the matched-dose ex vivo comparison by Al-Haj Husain et al. [6] assessed simulated tasks in extracted teeth rather than diagnostic decisions in patients. Neither finding establishes routine superiority of PCCT for endodontic indications. Selection should favor an appropriately optimized small-field CBCT examination when it answers the clinical question without additional radiation exposure.

The narrative review by Zanon et al. summarized these findings and emphasized that PCCT can achieve voxel sizes approaching 100 μm while simultaneously improving soft-tissue contrast and reducing image noise [1]. These technological characteristics may substantially improve early diagnosis and treatment planning in restorative dentistry and endodontics.

#### 3.1.2. Visualization of Periodontal Structures

High spatial resolution is important for visualization of periodontal osseous structures and the periodontal ligament space. Available PCCT studies have demonstrated favorable visualization of cortical and cancellous bone structures and periodontal ligament spaces [7], while comparative studies have reported improved image quality and dimensional accuracy relative to conventional imaging techniques [4,7]. However, direct diagnostic validation of PCCT for periodontal defect detection against histology or micro-CT is currently lacking. Therefore, although these imaging characteristics may be relevant to periodontal assessment, improved diagnostic accuracy for periodontal disease has not yet been established.

#### 3.1.3. Visualization of Trabecular Bone Architecture

The high spatial resolution of PCCT enables detailed visualization of cancellous and trabecular bone structures. Ruetters et al. [7] demonstrated detailed visualization of cortical and spongious bone, while Sawall et al. [8] reported higher dose-normalized contrast-to-noise ratios for dentin–bone interfaces with PCCT compared with DVT. However, these findings primarily reflect qualitative image quality and contrast-based performance. Direct validation of trabecular microarchitectural parameters, such as bone volume fraction (BV/TV) or trabecular thickness (Tb.Th), against histology or micro-CT has not been demonstrated in the cited oral and maxillofacial studies. Given that trabecular dimensions may approach the spatial-resolution limits of current PCCT acquisitions, its ability to provide accurate quantitative assessment of trabecular microarchitecture remains to be established.

Al-Haj Husain et al. [9] compared PCCT and CBCT for detecting simulated mandibular osseous lesions, including cystic lesions, in an ex vivo study using porcine mandibles. PCCT demonstrated higher lesion detectability scores, particularly at lower radiation doses. However, these findings require further validation in clinical patients with actual odontogenic lesions.

#### 3.1.4. Implant Planning Accuracy

Successful implant planning requires accurate assessment of alveolar ridge morphology, cortical thickness, bone density, and proximity to critical anatomical structures such as the mandibular canal and maxillary sinus floor.

**Figure 5:**
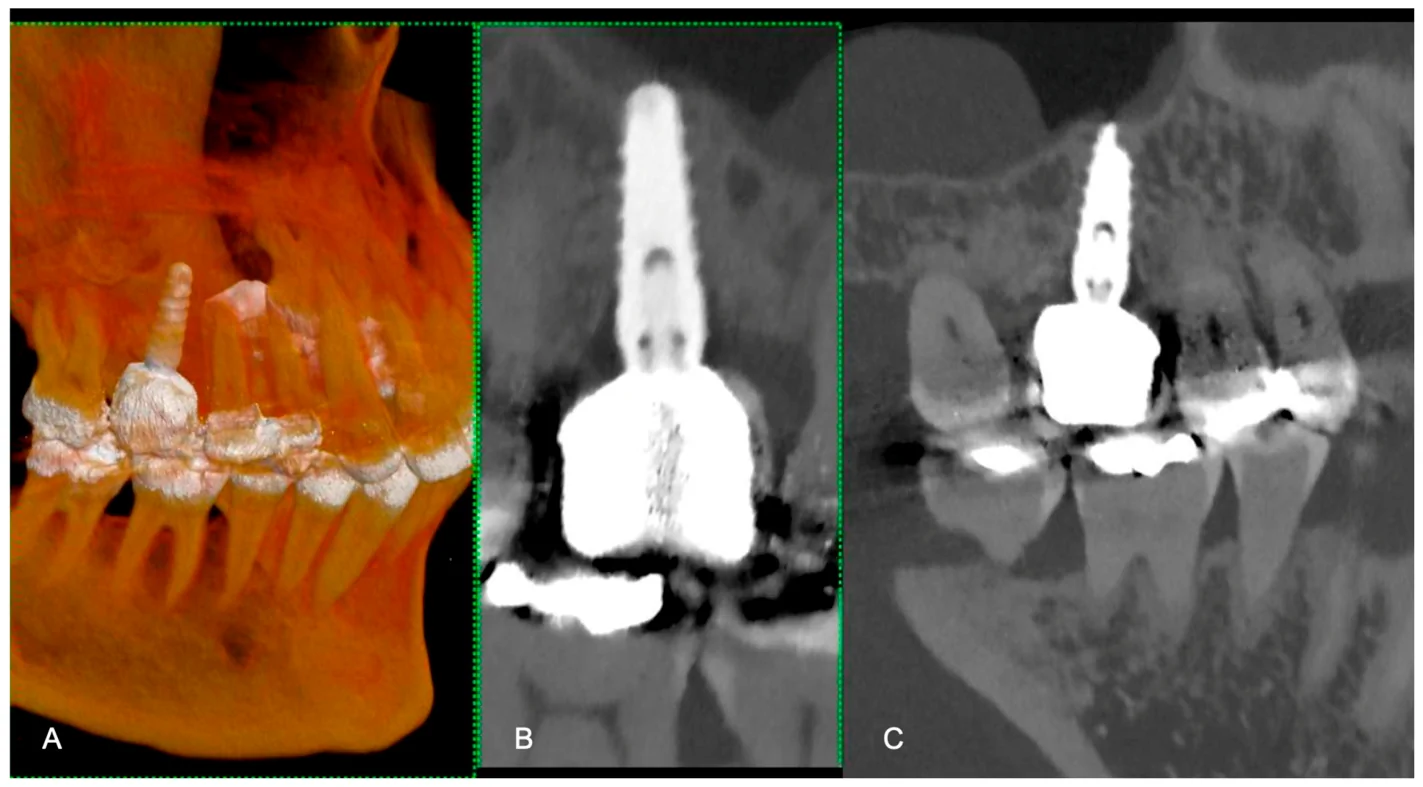
Photon-counting CT visualization of dental implants for implant planning. (**A**) Three-dimensional cinematic rendering of dental implants; (**B**,**C**) cross-sectional images demonstrating implant position, surrounding bone, and the relationship with adjacent anatomical structures. The images illustrate the high spatial resolution achievable with PCCT for implant assessment and treatment planning. Reproduced from Ref. [1].

PCCT has demonstrated substantial advantages for implant planning because of its high spatial resolution and improved artifact reduction [1,10]. Al-Haj Husain et al. demonstrated that ultra-high-resolution reconstructions and virtual monoenergetic imaging at 120–130 keV provided optimal image quality for implant visualization with significantly reduced metal artifacts (Figure 5) [10].

Their study showed improved delineation of implant screw threads and peri-implant cortical bone compared with conventional reconstructions [10]. Furthermore, reduced artifact burden improved evaluation of peri-implant osseous structures and adjacent anatomy.

Ruetters et al. also demonstrated that PCCT enabled highly detailed assessment of peri-implant structures and surrounding trabecular bone, suggesting potential advantages for preoperative implant planning and postoperative implant evaluation [7].

Zanon et al. highlighted that the combination of spectral imaging, reduced noise, and ultra-high-resolution imaging may improve surgical precision and decrease complications during implant placement [1].

These implant-related observations primarily demonstrate anatomic visibility and artifact reduction, not prospectively validated gains in implant placement accuracy, complication rates, or peri-implantitis diagnosis. PCCT should not replace CBCT for routine implant planning solely on the basis of sharper images; a reasonable indication would require a defined diagnostic problem that cannot be adequately addressed by conventional dental imaging.

### 3.2. Implantology and Metal Artifact Reduction

#### 3.2.1. Reduced Streak Artifacts

Metal artifacts caused by implants, crowns, amalgam restorations, and endodontic materials represent one of the major limitations of CBCT imaging [1,10]. Beam hardening and photon starvation frequently generate streak artifacts that obscure adjacent anatomical structures and reduce diagnostic confidence.

The magnitude and pattern of metal-related artifacts depend partly on the composition of dental implants and restorative materials. In the ex vivo study by Al-Haj Husain et al. [10], titanium-based dental implants were evaluated. In other clinical studies evaluating dental hardware and multiple dental implants, the specific composition of individual restorative materials or implants was not consistently reported.

**Figure 6:**
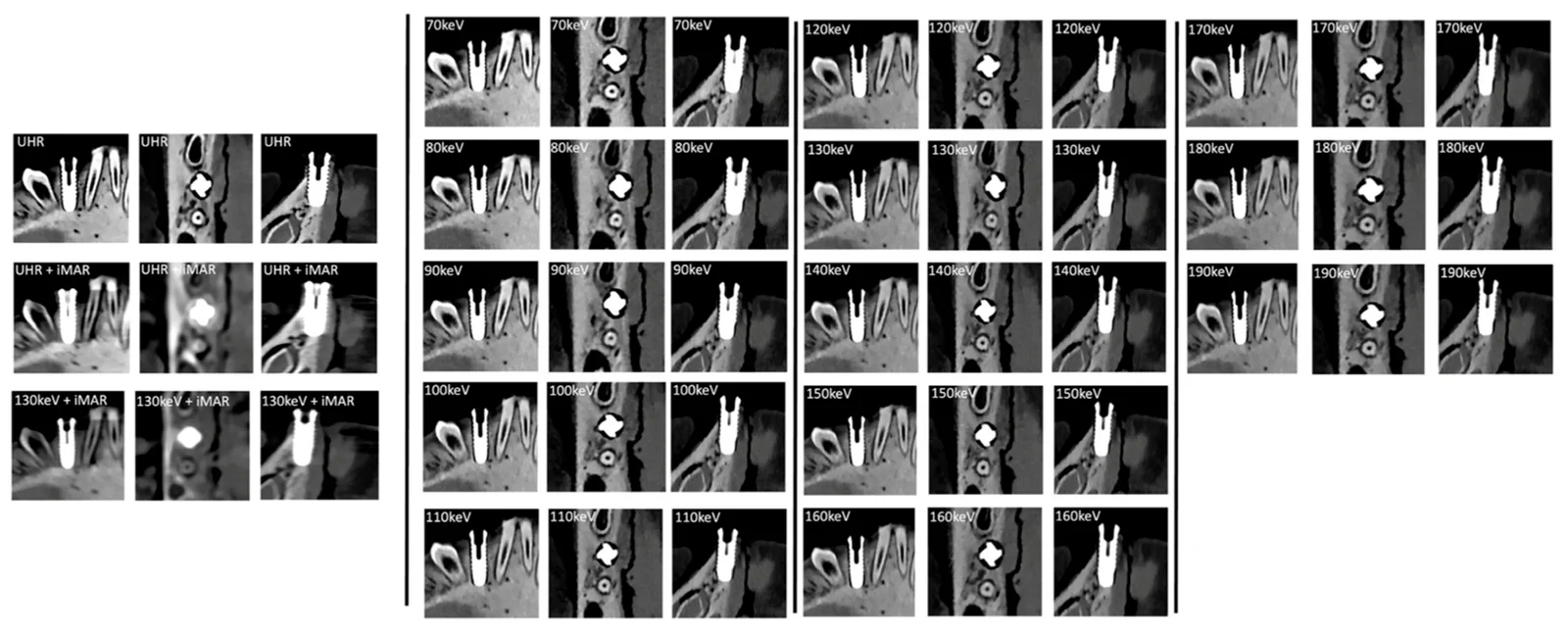
Representative visualization of a titanium-based dental implant using different photon-counting detector CT reconstruction techniques. The smaller block on the left shows, from top to bottom, ultra-high-resolution (UHR) images without iterative metal artifact reduction (iMAR), UHR images with iMAR, and virtual monoenergetic images (VMI) at 130 keV with iMAR. The larger block on the right shows VMI reconstructions from 70 to 190 keV at 10-keV increments. Each reconstruction is displayed in three corresponding imaging planes. UHR images without iMAR provide excellent visualization of the implant screw threads and bone–implant interface. With increasing VMI energy, metal-related artifacts progressively decrease, with VMI at 120–130 keV providing the optimal balance between image quality and artifact reduction. The addition of iMAR to UHR images or VMI at 130 keV did not further improve image quality. Reproduced from Ref. [10].

PCCT can substantially reduce metal-related artifacts through spectral imaging and virtual monoenergetic reconstructions [1,10]. Al-Haj Husain et al. demonstrated that VMI reconstructions at 120–130 keV substantially reduced artifacts from titanium-based dental implants while maintaining excellent image quality (Figure 6) [10]. Quantitative artifact burden progressively decreased at higher monoenergetic levels, with optimal performance observed at 120–130 keV [10].

Zanon et al. reviewed multiple studies demonstrating that VMI and iterative metal artifact reduction (iMAR) techniques significantly improve visualization around metallic dental hardware [1]. The review specifically cited studies by Risch et al., Layer et al., and Pallasch et al., which showed substantial reductions in artifact index measurements using high-keV monoenergetic reconstructions [1].

#### 3.2.2. Better Peri-Implant Bone Assessment

Accurate assessment of peri-implant bone is essential for evaluating osseointegration, peri-implantitis, fenestration, and peri-implant osteolysis.

**Figure 7:**
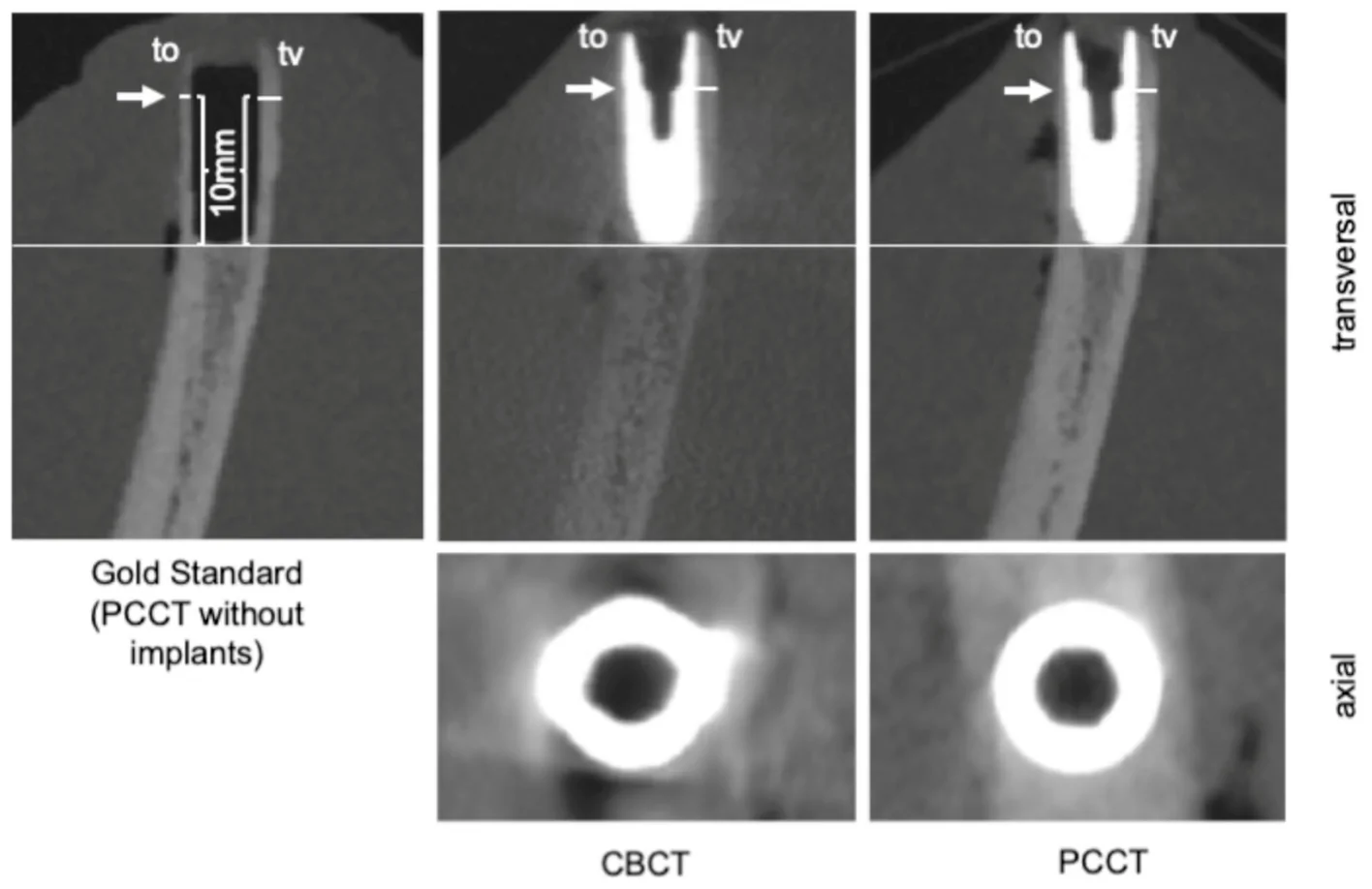
Comparison of peri-implant imaging with photon-counting CT (PCCT) and cone-beam CT (CBCT). Registered images demonstrate superior image quality and lower artifact burden with PCCT. The oral bone lamella remains identifiable at the same location in the gold-standard and PCCT images, whereas it is not assessable on CBCT because of implant-related artifacts. The reduced artifact burden with PCCT also permits visualization of individual implant threads and more precise measurement of vestibular bone thickness [7].

PCCT provides superior delineation of peri-implant cortical and cancellous bone structures because of its improved spatial resolution and artifact reduction capability [7,10]. Ruetters et al. demonstrated excellent visualization of peri-implant structures and surrounding bone using ultra-high-resolution PCCT imaging (Figure 7) [7,10].

Similarly, Al-Haj Husain et al. showed that PCCT enabled detailed visualization of the bone–implant interface and implant screw morphology while significantly reducing metal-induced artifacts [10]. Their study demonstrated that ultra-high-resolution reconstructions combined with sharp kernels produced optimal peri-implant image quality [10].

These improvements may facilitate earlier detection of peri-implant complications and improve long-term implant follow-up assessment.

A clinically meaningful comparison must begin with the indication and an optimized reference protocol. For routine endodontic evaluation, dentoalveolar assessment, and implant planning, small-field CBCT is generally more accessible and less costly; high-resolution CBCT may also equal or outperform PCCT for particular findings, including root cracks in Fontenele et al. [5]. PCCT could offer additional value when both detailed osseous anatomy and soft-tissue or spectral information are needed, or when metal artifacts preclude confident assessment. However, these are candidate indications rather than established superiority claims. Comparative studies should report sensitivity, specificity, diagnostic agreement with appropriate clinical or surgical reference standards, impact on patient management, and rates of avoidable repeat examinations, rather than relying solely on image-quality scores.

#### 3.2.3. Improved Visualization near Restorations

Visualization adjacent to metallic restorations remains challenging with conventional CBCT because of severe beam-hardening artifacts [1]. PCCT improves visualization near restorations through reduced electronic noise, spectral imaging capability, and advanced reconstruction algorithms [1,10]. Zanon et al. reviewed the use of virtual monoenergetic imaging and metal artifact reduction techniques for improving visualization around dental restorations and implants [1]. Vanden Broeke et al., discussed in the Zanon review, reported reduced artifact burden with spectral PCCT, while overall visualization quality remained comparable with CBCT [1]. In addition, Al-Haj Husain et al. demonstrated that high-keV virtual monoenergetic reconstructions substantially reduced metal-related artifacts from titanium-based dental implants [10]. These improvements may enhance diagnostic confidence and reduce the need for repeat imaging examinations in patients with extensive restorative dental work.

### 3.3. Comparison with CBCT in Dental Imaging

CBCT remains the most widely used modality in dental imaging because of its relatively low radiation dose and high spatial resolution for dentoalveolar structures [1,4]. Small field-of-view CBCT acquisitions provide adequate image quality for routine implant planning, orthodontics, and endodontics. Also, CBCT systems are broadly available in dental offices and outpatient imaging centers worldwide and remain more cost-effective and accessible than PCCT systems [1].

**Figure 8:**
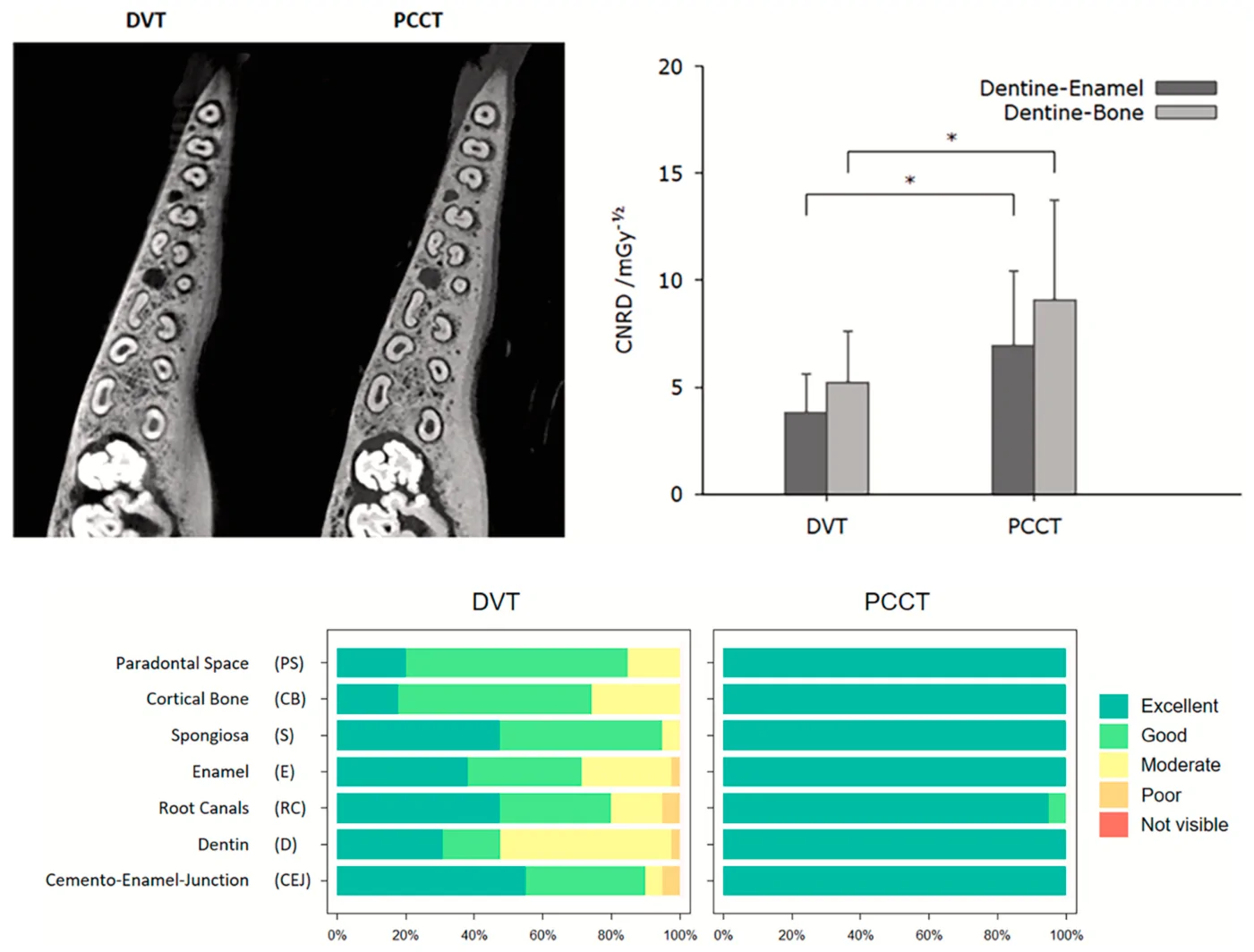
Comparison of dental imaging performance between digital volume tomography (DVT/CBCT) and photon-counting CT (PCCT). The upper panel shows representative DVT and PCCT reconstructions and dose-normalized contrast-to-noise ratios for dentin–enamel and dentin–bone interfaces; the lower panel shows qualitative image-quality scores for diagnostically relevant dental structures. PCCT demonstrated superior image quality and dose efficiency despite acquisition at approximately one-quarter of the DVT dose. An asterisk (*) indicates statistical significance (paired *t*-test, *p* < 0.05). Reproduced from Ref. [8].

In an ex vivo study using porcine jaws, Sawall et al. [8] demonstrated that a clinical PCCT scanner achieved superior image quality compared with digital volume tomography (DVT) at approximately one-quarter of the DVT CTDI under the evaluated acquisition protocols (Figure 8) [8]. These findings suggest that optimized PCCT protocols may eventually challenge CBCT dose advantages in selected applications. Zanon et al. emphasized that PCCT offers superior soft tissue visualization compared with conventional CBCT systems, potentially improving evaluation of adjacent soft tissue anatomy [1].

Rau et al. also demonstrated superior delineation of fine dental anatomy and accessory canals using PCCT compared with CBCT [4]. One of the major advantages of PCCT is spectral imaging capability. Virtual monoenergetic imaging enables optimization of image contrast and artifact suppression using different energy reconstructions from a single acquisition dataset [1,10]. This capability is unavailable in conventional CBCT systems and represents one of the major technological advances of PCCT. PCCT substantially reduces metal-induced artifacts through spectral imaging and advanced reconstruction algorithms [1,10]. Multiple studies reviewed by Zanon et al. evaluated VMI and metal artifact reduction techniques for reducing artifacts near metallic restorations and implants [1]. Al-Haj Husain et al. further demonstrated that VMI at 120–130 keV significantly reduced implant-related artifact burden while preserving excellent image quality, allowing improved peri-implant visualization [10].

Nevertheless, currently the widespread availability, low cost, and rapid acquisition continue to make CBCT the preferred modality for standard dental imaging applications [1]. Beyond image quality and radiation dose, several practical factors should be considered when comparing PCCT with CBCT. Dedicated dental CBCT systems offer adjustable fields of view tailored to specific dentoalveolar regions, whereas clinical PCCT systems are primarily designed for broader diagnostic applications, making careful scan-range optimization essential [11,12]. Acquisition time and susceptibility to patient motion are also important considerations, particularly for examinations requiring visualization of fine dental structures. Motion-related artifacts are well documented in dental CBCT, although direct comparative evidence establishing differences in motion sensitivity between PCCT and CBCT remains limited [13]. Furthermore, clinical PCCT examinations are generally performed with patients in the supine position, whereas dental CBCT systems may accommodate seated, standing, or supine positioning, depending on the equipment [12,14]. These practical differences should be considered when selecting the most appropriate modality for individual clinical indications.

A comparative summary of the principal technical and clinical characteristics of CBCT, EID-CT, and PCCT is provided in Table 1.

### 3.4. Radiation Dose and Protocol Optimization

Radiation dose is an important consideration in oral and maxillofacial imaging, particularly in pediatric patients and individuals requiring repeated examinations. PCCT has been investigated as a potentially dose-efficient imaging technique; however, the available evidence indicates that radiation exposure depends on the clinical indication, anatomical region, acquisition parameters, spatial-resolution requirements, and comparator modality. Therefore, dose performance should be interpreted in the context of the specific imaging task and acquisition protocol rather than as an inherent advantage of PCCT across all oral and maxillofacial applications.

Available dental studies illustrate this protocol dependence. Fontenele et al. [5] reported an estimated effective dose of 0.85 mSv for the PCCT protocol used for challenging endodontic diagnostic tasks, compared with 0.03–0.13 mSv for standard-resolution CBCT and 0.01–0.04 mSv for high-resolution CBCT. In contrast, Sawall et al. [8] demonstrated superior dental image quality with clinical PCCT at approximately one-quarter of the radiation dose of DVT. These findings indicate that the relative dose performance of PCCT and CBCT/DVT varies according to the acquisition protocol and diagnostic task.

Evidence from pediatric imaging further demonstrates the potential for dose optimization. Lee et al. [15] compared PCCT with conventional energy-integrating detector CT in 110 pediatric temporal bone examinations and reported an overall radiation-dose reduction of approximately 43%, with reductions exceeding 70% in children younger than six years. In a multi-institutional study including 4772 pediatric patients undergoing 6464 PCCT examinations, Habib Geryes et al. [16] provided contemporary pediatric dose benchmarks and reported that protocols were adapted according to anatomical region and patient weight.

Dose-efficient PCCT has also been investigated in sinonasal imaging. Grunz et al. [17] demonstrated diagnostically adequate ultra-low-dose PCCT of the paranasal sinuses at radiation exposures as low as 0.08 mGy. Kaatsch et al. [18] compared ultra-low-dose PCCT with CBCT for paranasal sinus imaging and reported superior subjective image quality and anatomical delineation with PCCT, with a mean effective radiation dose approximately one-quarter of that observed with CBCT. In postoperative maxillofacial imaging, Al-Haj Husain et al. [19] also demonstrated the feasibility of PCCT assessment of fracture reduction and osteosynthesis materials under low-dose conditions.

The available literature does not currently provide a single standardized PCCT acquisition protocol for dental and maxillofacial imaging. Instead, acquisition and reconstruction parameters vary among studies according to the anatomical region, patient characteristics, diagnostic task, required spatial resolution, and dose considerations. Accordingly, the term “standard PCCT protocol,” when used in individual studies, should be interpreted as the routine clinical acquisition protocol employed in that particular study rather than as a universally standardized protocol for oral and maxillofacial imaging. Further comparative studies are required to establish task-specific acquisition and reconstruction protocols and appropriate dose benchmarks for dental and maxillofacial applications. To facilitate interpretation of the available evidence, Table 2 summarizes the study designs, sample characteristics, imaging platforms, radiation-dose parameters, comparators, and principal findings of representative investigations of PCCT in dental and maxillofacial imaging. Importantly, a substantial proportion of the available evidence originates from phantom, ex vivo, cadaveric, or simulated-disease models. Although these investigations demonstrate technical feasibility and improvements in selected image-quality metrics, their findings cannot be directly extrapolated to diagnostic accuracy, clinical decision-making, or patient outcomes without further validation.

At present, there is no widely validated, standardized set of indication-specific PCCT protocols for oral and maxillofacial imaging. Future reporting should specify scanner and detector mode, kVp and spectral settings, tube current/exposure, tin filtration when used, field of view and z-axis coverage, reconstruction kernel and thickness, iterative or metal-artifact reduction settings, and dosimetry methodology. Optimization must be task-specific, with standardized diagnostic acceptability thresholds, organ-dose assessment where relevant, and prospective comparison against contemporary optimized CBCT—not against arbitrarily high-dose reference protocols.

PCCT dose efficiency is a property of a detector and acquisition chain, not a guarantee that every clinical PCCT examination delivers a lower effective dose than CBCT. Cross-study comparisons of CTDI or dose-normalized contrast-to-noise ratio cannot be treated as equivalent to patient effective-dose or diagnostic-performance comparisons; reported CTDI values, effective doses, scan ranges, and imaging tasks must be kept distinct. The existing dental comparisons are notably discordant: Fontenele et al. [5] reported 0.85 mSv for their PCCT protocol versus 0.01–0.13 mSv for the assessed CBCT protocols, while Al-Haj Husain et al. [6] tested approximately dose-matched ex vivo protocols, and Sawall et al. [8] reported favorable PCCT image quality at lower CTDI than the selected DVT comparison. These results are not interchangeable or generalizable to all devices.

### 3.5. Maxillofacial Trauma

The previously discussed PCCT advantages have demonstrated considerable potential in the evaluation and management of maxillofacial trauma, in particular, the improved visualization of osseous structures adjacent to metallic hardware is particularly relevant in oral and maxillofacial trauma imaging, where accurate delineation of fracture lines, osteosynthesis materials, and surrounding anatomical structures is essential for diagnosis, surgical planning, and postoperative follow-up.

One of the most significant advantages of PCCT in trauma imaging is its enhanced ability to detect subtle fractures and microfractures. Compared with CBCT and energy-integrating detector CT (EID-CT), PCCT may improve visualization of cortical bone detail and fracture morphology owing to its smaller detector elements and higher spatial resolution. Klintström et al. [20] demonstrated that PCCT achieved significantly higher signal-to-noise ratios and superior bone sharpness compared with CBCT in a cadaveric maxillofacial model, resulting in improved delineation of mandibular and orbital structures important for trauma assessment. The authors further noted that the improved anatomical visualization may support more accurate diagnosis and management of facial fractures, particularly in anatomically complex regions.

In addition to improved fracture visualization, PCCT has shown advantages in the assessment of postoperative fracture fixation and osteosynthesis materials. Al-Haj Husain et al. [19] evaluated ultra-high-resolution PCCT in comparison with CBCT for postoperative imaging of mandibular fractures stabilized using titanium and bioresorbable fixation systems. The study demonstrated that PCCT enabled excellent visualization of fracture reduction and implant positioning, with superior reproducibility and measurement accuracy compared with CBCT, even under low-dose conditions. Importantly, the authors reported that PCCT achieved more precise quantitative measurements of fracture gaps and osteosynthesis materials relative to ground-truth values, highlighting its utility for postoperative assessment and surgical quality control.

Similarly, Sandhu et al. [21] compared PCCT and CBCT in simulated mandibular trauma and found that PCCT consistently provided superior overall image quality, improved visualization of fracture lines, and better delineation of peri-osteosynthetic structures. The reduction in metal-related artifacts observed with PCCT was particularly beneficial in cases involving titanium reconstruction plates, where conventional imaging techniques often suffer from beam-hardening and streak artifacts that obscure adjacent anatomy. The authors concluded that PCCT may offer significant diagnostic advantages in perioperative maxillofacial trauma imaging, especially in younger patients who may require repeated imaging examinations.

The high-resolution datasets generated by PCCT also support advanced surgical planning and digital workflow integration. Kalaitsidou et al. [22] demonstrated the feasibility of incorporating PCCT into virtual surgical planning (VSP) and CAD/CAM-based workflows for complex maxillofacial reconstruction. The authors reported that PCCT enabled detailed visualization of cortical and medullary bone, improved assessment of osseous involvement, and reduced metal artifacts through spectral reconstructions. Moreover, the generated datasets were fully compatible with contemporary VSP software platforms, facilitating accurate segmentation and multi-segment osteotomy planning. Although the study primarily focused on reconstructive surgery, these findings are highly relevant to maxillofacial trauma, where precise three-dimensional visualization and computer-assisted planning are increasingly important for the management of complex fractures and secondary reconstructions.

Another clinically important advantage of PCCT in trauma imaging is its potential for dose-efficient imaging. Maxillofacial trauma patients frequently undergo multiple imaging examinations during diagnosis, intraoperative guidance, and postoperative follow-up. The enhanced dose efficiency and reduced electronic noise of PCCT allow acquisition of diagnostically acceptable images at lower radiation doses compared with conventional CT systems. This is especially relevant in younger trauma populations, where minimizing cumulative radiation exposure remains a major consideration [20].

### 3.6. Temporomandibular Joint Disorders

Although direct clinical evidence specifically evaluating PCCT in temporomandibular joint (TMJ) disorders remains limited, existing PCCT studies in maxillofacial imaging demonstrate improved visualization of fine osseous anatomy and provide a technical rationale for its potential application to TMJ assessment [20]. High-resolution imaging of the mandibular condyle is important for the assessment of condylar morphology, degenerative joint disease, remodeling changes, and other osseous abnormalities associated with TMJ disorders.

A potential benefit of PCCT in TMJ imaging is its ability to provide highly detailed visualization of cortical and trabecular bone architecture. The smaller detector elements and improved spatial resolution of PCCT enable detailed depiction of fine osseous structures compared with conventional energy-integrating detector CT systems [20]. These technical characteristics could potentially improve evaluation of condylar morphology, including cortical irregularities, flattening, erosions, osteophyte formation, and subchondral sclerosis. However, the diagnostic performance of PCCT for detecting these specific TMJ abnormalities has not yet been established in dedicated clinical studies.

Another important application of advanced imaging in TMJ disorders is the assessment of condylar growth abnormalities and asymmetry. Ouyang et al. (2023) [23] evaluated condylar growth activity and three-dimensional mandibular growth patterns in patients with unilateral condylar hyperplasia using SPECT and CT-based measurements. The authors demonstrated that three-dimensional CT analysis allowed detailed assessment of condylar morphology and mandibular growth changes, including increases in ramus height and diagonal mandibular growth in active disease. Although this study did not specifically investigate PCCT, its findings emphasize the importance of high-resolution three-dimensional imaging for evaluating condylar morphology and growth abnormalities. Given the high spatial resolution demonstrated with PCCT in maxillofacial imaging [20], its potential role in the assessment of subtle condylar abnormalities warrants dedicated investigation.

PCCT may also have potential for the assessment of osseous changes associated with TMJ osteoarthritis. Its improved spatial resolution and reduced electronic noise could theoretically facilitate visualization of cortical erosions, subchondral changes, trabecular alterations, and osteophytes. Nevertheless, direct evidence demonstrating improved detection or diagnostic accuracy for TMJ osteoarthritis with PCCT is currently lacking, and this potential application should therefore be considered hypothesis-generating rather than an established clinical indication.

Additionally, the spectral capabilities of PCCT may potentially improve characterization of tissues and materials around the TMJ. Multi-energy datasets and virtual monoenergetic reconstructions can reduce beam-hardening and metal-related artifacts in maxillofacial imaging [24]. This capability may be particularly relevant to TMJ assessment in patients with dental restorations or other metallic materials near the region of interest. However, dedicated studies are required to determine whether these technical advantages translate into improved diagnostic performance for TMJ disorders.

MRI remains the reference imaging modality for evaluating the articular disc and other soft-tissue components of the temporomandibular joint. Accordingly, PCCT should not be considered a replacement for MRI in the assessment of internal derangement. Its potential role in TMJ imaging is primarily related to the evaluation of osseous structures, although dedicated clinical studies are required to establish its diagnostic performance in TMJ disorders [25].

### 3.7. Oncologic Imaging and Head and Neck Cancer Applications

Photon-counting detector CT may provide important advantages in oncologic imaging of the oral and maxillofacial region, particularly in patients with head and neck squamous cell carcinoma (HNSCC). In addition to its high spatial resolution, the intrinsic spectral capabilities of PCCT enable virtual monoenergetic imaging (VMI), iodine quantification, and material-specific reconstructions, potentially improving tumor conspicuity, tissue characterization, and local staging [26,27].

Virtual monoenergetic reconstructions can optimize contrast and image quality in head and neck imaging. Farhadi et al. evaluated VMI derived from clinical photon-counting CT datasets and demonstrated energy-dependent improvements in signal-to-noise ratio and subjective image quality, with preferred energy levels varying according to the diagnostic task [26]. These findings support the potential role of spectral PCCT reconstructions in improving visualization of anatomically complex head and neck structures.

**Figure 9:**
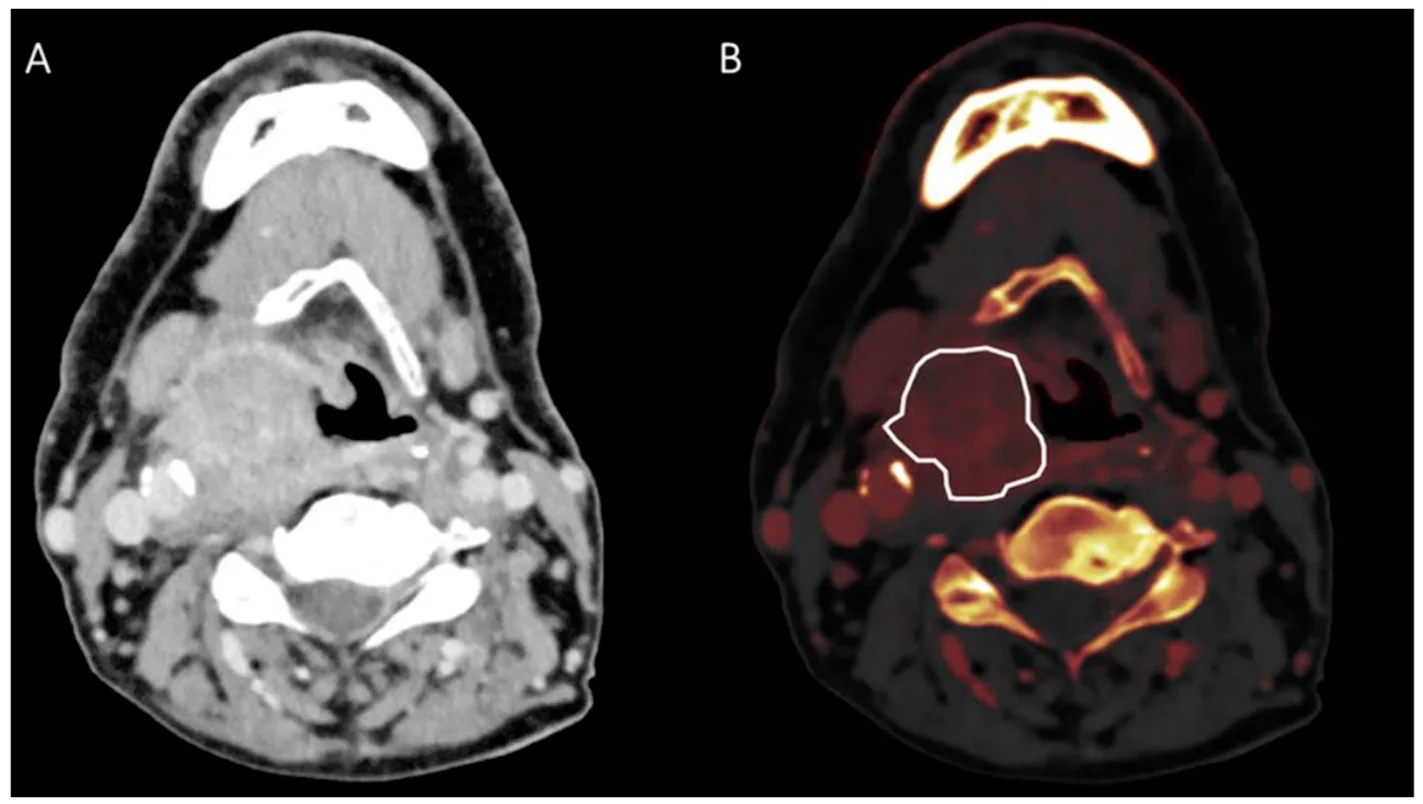
Representative photon-counting CT findings in a patient with grade 2 T4N1M0 head and neck squamous cell carcinoma. (**A**) CT image demonstrating a large T4 oropharyngeal tumor. (**B**) Iodine map with a region of interest, outlined in white, placed along the tumor margins, showing an iodine concentration of 1.7 mg/mL and a normalized iodine concentration of 0.65. Reproduced from Ref. [27].

The quantitative spectral information provided by PCCT may also offer imaging biomarkers related to tumor biology. Surov et al. evaluated 84 patients with untreated oropharyngeal squamous cell carcinoma and demonstrated excellent interreader reproducibility of normalized intratumoral iodine concentration (NIC; ICC = 0.96). NIC was significantly associated with tumor cellularity, nodal status, and HPV status, suggesting that iodine mapping may provide complementary information beyond conventional morphological assessment in oropharyngeal tumors (Figure 9) [27].

PCCT may additionally have a role in radiotherapy planning for head and neck malignancies. Lamaj et al. investigated virtual monoenergetic and relative electron density reconstructions derived from PCCT for treatment planning and dose calculation in patients with head and neck cancer. Treatment plans based on PCCT reconstructions showed clinically feasible dosimetric performance, while relative electron density images offered the additional advantage of not requiring scanner-specific calibration [28].

These characteristics may be particularly relevant in oral and maxillofacial oncology, where accurate delineation of tumors adjacent to bone, teeth, and metallic dental materials can be challenging. Overall, PCCT has the potential to expand CT assessment of head and neck malignancies from conventional morphological imaging toward combined high-resolution, spectral, and quantitative tumor characterization. However, current clinical evidence remains limited, and larger prospective studies are required to determine whether these technical and quantitative advantages translate into improved staging accuracy, treatment planning, and clinical outcomes [26,27,28].

### 3.8. Pediatric Oral and Maxillofacial Applications

Imaging plays an important role in the diagnosis and treatment planning of childhood malocclusion, particularly for the assessment of dentofacial morphology and associated dental abnormalities [29]. The improved dose efficiency of photon-counting detector CT may be particularly important in pediatric oral and maxillofacial imaging, because children have greater radiosensitivity and a longer lifetime during which radiation-related effects may occur. At the same time, pediatric head and neck imaging frequently requires visualization of small and densely packed anatomical structures, making the combination of high spatial resolution and dose efficiency provided by PCCT particularly attractive [15,16].

Clinical evidence from pediatric temporal bone imaging demonstrates the potential of PCCT for high-resolution, low-dose assessment of small craniofacial structures. Lee et al. compared PCCT with conventional energy-integrating detector CT in 110 pediatric temporal bone examinations and found significantly higher image-quality scores with PCCT for the tympanic membrane, incudostapedial joint, stapedial crura, and cochlear modiolus, as well as superior overall image quality. Importantly, PCCT was associated with an overall radiation-dose reduction of approximately 43%, with reductions exceeding 70% in children younger than six years [15].

Broader pediatric experience further supports the feasibility of dose-optimized PCCT in routine clinical practice. In a multi-institutional study, Habib Geryes et al. evaluated 4772 pediatric patients undergoing 6464 PCCT examinations across three institutions. Protocols were adapted according to anatomical region and patient weight, providing contemporary pediatric dose benchmarks and demonstrating the feasibility of implementing PCCT across a wide range of clinical indications [16].

These characteristics may be particularly relevant to pediatric oral and maxillofacial indications requiring detailed assessment of thin osseous structures, including craniofacial abnormalities, maxillofacial trauma, sinonasal disease, and selected temporal bone disorders. Nevertheless, direct evidence specifically evaluating PCCT for pediatric dental and dentoalveolar indications remains limited. Future studies should directly compare PCCT with CBCT for specific pediatric oral and maxillofacial applications and determine whether the combination of improved spatial resolution, spectral information, and dose efficiency provides a clinically meaningful advantage.

Adherence to the ALARA (As Low As Reasonably Achievable) and ALADA (As Low As Diagnostically Acceptable) principles is particularly important in pediatric imaging. Low-dose CBCT remains an established modality for routine orthodontic and dentoalveolar imaging [1,29]. Although pediatric PCCT studies have demonstrated the feasibility of dose-optimized imaging, the available dose-reduction evidence should be interpreted according to the specific anatomical region and comparator modality. In particular, Lee et al. reported substantial dose reductions for pediatric temporal bone imaging compared with conventional EID-CT, rather than CBCT [15]. Therefore, dedicated studies directly comparing PCCT with CBCT are required before dose or diagnostic advantages can be established for pediatric dental and dentoalveolar applications.

### 3.9. Paranasal Sinus Imaging

PCCT advantages are particularly valuable in chronic sinusitis assessment, osteomeatal complex visualization, and pre-surgical planning for functional endoscopic sinus surgery (FESS), where detailed depiction of delicate sinonasal anatomy is essential.

One of the principal clinical applications of PCCT in sinus imaging is the evaluation of chronic rhinosinusitis and inflammatory sinonasal disease. Conventional EID-CT has long been regarded as the reference standard for assessing mucosal thickening, sinus opacification, and osteomeatal obstruction; however, PCCT offers further improvements in image quality while simultaneously reducing radiation exposure. Grunz et al. [17] investigated ultra-low-dose PCCT imaging of the paranasal sinuses using tin prefiltration and demonstrated that diagnostically adequate imaging could be achieved at remarkably low radiation doses. The authors reported that PCCT protocols for acute rhinosinusitis evaluation were feasible at radiation exposures as low as 0.08 mGy while maintaining sufficient diagnostic quality. Compared with EID-CT, PCCT consistently achieved superior image quality at lower dose levels, particularly for visualization of sinonasal anatomy and inflammatory changes. These findings highlight the substantial dose-reduction potential of PCCT in routine sinus imaging, especially for patients requiring repeated follow-up examinations.

The improved spatial resolution of PCCT is particularly advantageous for visualization of the osteomeatal complex and intricate sinonasal anatomy. Accurate depiction of structures such as the frontal recess, uncinate process, lamina papyracea, lamina cribrosa, and sphenoethmoidal recess is essential for both diagnosis and surgical planning. Kaatsch et al. [18] compared ultra-low-dose PCCT with CBCT in paranasal sinus imaging and demonstrated significantly superior subjective image quality and anatomical delineation with PCCT. The authors reported that PCCT provided better visualization of critical sinonasal structures, including the frontal recess, lamina cribrosa, and osteomeatal region, while also achieving significantly higher contrast-to-noise ratios and lower radiation exposure than CBCT. Mean effective radiation dose with PCCT was approximately one quarter of that observed with CBCT, emphasizing its potential as a dose-efficient alternative for sinonasal imaging.

Beyond diagnostic imaging, PCCT demonstrates major advantages in pre-surgical planning and image-guided surgery. Functional endoscopic sinus surgery requires highly accurate visualization of thin osseous boundaries and adjacent critical structures, including the skull base, orbit, optic nerve canal, and internal carotid artery. Ernst et al. [30] evaluated the performance of PCCT in image-guided sinus surgery and demonstrated significantly improved intraoperative navigation accuracy compared with dual-energy and spectral detector CT systems. The study showed that PCCT maintained accurate image-guided surgery registration even at ultra-low-dose settings, whereas conventional systems exhibited progressive inaccuracies at lower radiation exposures. Importantly, the superior spatial resolution and contrast-to-noise ratio of PCCT enabled enhanced visualization of critical anatomical landmarks and improved surgical navigation precision.

The inherent spectral imaging capability of PCCT further enhances sinonasal imaging by improving tissue characterization and reducing beam-hardening artifacts from dental restorations and metallic hardware. This is especially relevant in maxillofacial and sinonasal imaging, where metallic dental restorations frequently degrade image quality in adjacent anatomical regions. 

Another important advantage of PCCT in airway and sinus imaging is the possibility of repeated low-dose examinations with preserved diagnostic quality. Chronic rhinosinusitis patients often undergo serial imaging for disease monitoring, postoperative assessment, or surgical planning. The improved dose efficiency of PCCT therefore offers important long-term clinical benefits by minimizing cumulative radiation exposure while preserving high-quality anatomical visualization.

### 3.10. Quantitative Imaging and Biomarkers

PCCT has significant potential in quantitative imaging and biomarker development due to its high spatial resolution, spectral imaging capability, and improved quantitative stability. These properties enable accurate assessment of bone mineral density (BMD), advanced material decomposition, and emerging applications in artificial intelligence (AI)-based radiomics and longitudinal biomarker analysis.

One of the most promising applications of PCCT is quantitative bone mineral density assessment. Conventional dual-energy X-ray absorptiometry (DXA) remains the clinical standard for osteoporosis screening; however, DXA provides only two-dimensional areal density measurements and lacks information regarding trabecular microarchitecture and volumetric bone density. Quantitative CT (QCT) and high-resolution peripheral QCT (HR-pQCT) offer improved three-dimensional characterization but are limited by calibration requirements, restricted anatomical coverage, or lengthy acquisition times.

Quintiens et al. [31] investigated the use of photon-counting CT for quantitative volumetric bone mineral density (vBMD) assessment in the peripheral skeleton. PCCT-derived vBMD measurements showed a strong correlation with HR-pQCT measurements (Pearson r = 0.986). However, correlation does not establish agreement between measurement methods, and these findings should not be interpreted as validation of PCCT for quantitative assessment of alveolar or maxillofacial bone. Dedicated studies in the oral and maxillofacial region are required to establish its quantitative accuracy for these applications.

Ma et al. [32] investigated PCCT-derived virtual monoenergetic imaging for vBMD quantification in a rabbit tibial defect model, using micro-CT as the reference. The authors reported strong correlations between VMI-derived CT values and micro-CT-measured vBMD across reconstructed energy levels (r > 0.925), with the highest correlation at 50 keV (r = 0.951). However, because this study was performed in rabbit tibiae, these findings cannot be directly extrapolated to quantitative assessment of alveolar or maxillofacial bone.

The energy-resolved data acquired with PCCT enable virtual monoenergetic reconstructions at different energy levels from a single acquisition. In the experimental rabbit tibial model evaluated by Ma et al. [32], VMI-derived CT values showed strong correlations with micro-CT-derived vBMD. These findings support the potential of spectral PCCT for quantitative bone assessment, although validation in oral and maxillofacial applications remains necessary [32].

Material decomposition is another critical quantitative application enabled by PCCT. By discriminating photons according to energy, PCCT can differentiate materials with overlapping attenuation characteristics on conventional CT, such as calcium, iodine, soft tissue, and bone marrow components. This allows improved tissue characterization and enhanced biomarker analysis. In musculoskeletal imaging, material decomposition may permit more accurate separation of cortical bone, trabecular bone, calcifications, and surrounding soft tissues. Such capabilities are particularly valuable in osteoporosis evaluation, inflammatory arthropathies, bone marrow disorders, and assessment of implant integration.

Beyond bone density assessment, the high-resolution and spectral datasets generated by PCCT provide an excellent foundation for AI-driven radiomics and machine learning applications. Radiomics relies on extraction of quantitative imaging features such as texture, intensity distributions, spectral signatures, and microstructural patterns that may not be visually appreciable. The improved signal fidelity, reduced noise, and enhanced spatial resolution of PCCT may improve the robustness and reproducibility of radiomic biomarkers. Potential future applications include automated fracture risk prediction, detection of early osteoarthritis, assessment of tumor heterogeneity, evaluation of implant osseointegration, and prediction of treatment response.

Moreover, the improved consistency of attenuation values across acquisitions may help address one of the major limitations of radiomics research-poor reproducibility between scanners and protocols. Because PCCT produces more stable monoenergetic datasets with reduced variability related to acquisition parameters, it may facilitate the development of standardized radiomics pipelines suitable for multicenter AI studies and precision medicine applications.

## 4. Clinical Perspectives and Future Directions

Although the potential applications of PCCT in dental imaging have been previously discussed by Zanon et al. [1], recent comparative evidence [5,6,8] allows for a more critical appraisal of its potential clinical role. Taken together, these findings suggest that the value of PCCT should be assessed according to specific diagnostic indications rather than technical image-quality improvements alone. In particular, the available evidence highlights important differences between experimental imaging performance and clinically validated diagnostic benefits, as well as substantial variability in radiation exposure across acquisition protocols. These considerations support a more selective approach to clinical implementation, with PCCT potentially complementing CBCT in complex cases where spectral information, soft-tissue assessment, or metal-artifact reduction may offer additional value. However, prospective comparative studies, standardized indication-specific protocols, and cost-effectiveness analyses remain necessary before broader clinical adoption can be justified.

From a clinical perspective, PCCT is likely to emerge as a complementary rather than replacement modality for CBCT in oral and maxillofacial imaging. CBCT will probably remain the preferred technique for many routine dental applications because of its widespread availability, lower cost, and established clinical workflows, whereas PCCT may offer particular advantages in diagnostically challenging cases requiring ultra-high spatial resolution, improved soft-tissue contrast, spectral information, or effective metal artifact reduction [1,8,10]. Its improved dose efficiency may be especially relevant in pediatric patients and in individuals requiring repeated examinations, while high-resolution and spectral reconstructions may expand its role in implant assessment, complex craniofacial imaging, trauma, sinonasal disease, and postoperative evaluation [10,15,16,17,18,20,30]. In addition, the quantitative and energy-resolved information provided by PCCT may support emerging applications in material decomposition, bone-density assessment, radiomics, and AI-based image analysis [31,32].

Future developments in dental imaging should also consider advances in competing technologies. Recent studies have demonstrated that artificial intelligence-based denoising and iterative reconstruction techniques can improve CBCT image quality [33]. Furthermore, metal artifact reduction algorithms are being investigated to reduce artifacts associated with dental implants, although their effectiveness remains variable [34]. These developments may influence the relative clinical advantages of PCCT, highlighting the need for future comparative studies.

However, current evidence in oral and maxillofacial radiology remains limited, with many reported advantages of PCCT derived from phantom, ex vivo, animal, and small-scale feasibility studies rather than adequately powered clinical investigations. Consequently, improvements in technical endpoints, such as spatial resolution, image quality, artifact reduction, and quantitative measurement performance, should not be interpreted as established benefits in diagnostic accuracy or patient outcomes. Furthermore, the predominance of evidence obtained using a single commercially established PCCT vendor platform may limit the generalizability of findings to systems employing different detector technologies and acquisition geometries [35]. Future research should prioritize prospective multicenter comparisons with CBCT and conventional CT, addressing task-specific diagnostic performance, optimized low-dose protocols, reconstruction standardization, reproducibility, and cost-effectiveness. Practical considerations, including field-of-view optimization, acquisition time, motion sensitivity, and patient positioning, also warrant systematic evaluation. Such investigations are essential to establish the clinical indications in which PCCT provides meaningful added value and to support its broader integration into routine oral and maxillofacial imaging.

An indication-based implementation hierarchy follows from these limitations. Routine localized dental tasks should ordinarily remain within optimized conventional dental pathways, particularly CBCT when three-dimensional imaging is justified. PCCT merits clinical evaluation where there is a plausible incremental need for spectral characterization, combined bone and soft-tissue assessment, or severe metal-artifact challenges; even here, superiority in diagnostic accuracy and patient outcomes remains to be demonstrated. Pediatric and repeat imaging require an especially explicit justification of the examination and a task-appropriate dose benchmark.

For clinical translation, the key unresolved question is not whether PCCT can produce finer images, but whether the additional information improves diagnostic accuracy or influences treatment decisions at an acceptable radiation dose and financial cost. Importantly, existing phantom, ex vivo, animal, and preliminary clinical findings do not collectively establish superior diagnostic accuracy, improved patient outcomes, or lower radiation exposure compared with optimized CBCT across routine dental indications. The absence of standardized oral and maxillofacial PCCT protocols further limits reproducibility and generalizability. Future research should therefore prioritize prospective, adequately powered, task-matched PCCT–CBCT comparisons, blinded reader studies with prespecified reference standards, patient-level diagnostic endpoints, comprehensive dosimetry, and reproducibility assessments across scanner models and reconstruction settings. Economic evaluations should additionally address equipment availability, referral pathways, throughput, and downstream investigations. Phantom and ex vivo optimization remains valuable but cannot substitute for clinical validation.

## 5. Conclusions

Photon-counting detector CT represents a promising technological advancement for oral and maxillofacial radiology by combining ultra-high spatial resolution, improved dose efficiency, reduced electronic noise, and intrinsic spectral imaging. Current evidence highlights its potential for enhanced visualization of fine dental and osseous anatomy, endodontic structures, peri-implant bone, maxillofacial fractures, and sinonasal anatomy, while high-keV virtual monoenergetic imaging and other spectral reconstructions offer promising opportunities to reduce artifacts from dental restorations and metallic implants. PCCT may also expand the role of CT beyond anatomical assessment through quantitative applications such as bone mineral density measurement, material decomposition, and imaging biomarker development.

Despite these promising advances, CBCT remains the preferred modality for many routine dental applications because of its widespread availability, lower cost, and established clinical workflows. Although experimental and preliminary clinical studies have demonstrated encouraging technical and imaging performance, their findings require further clinical validation to establish whether these improvements translate into meaningful gains in diagnostic accuracy and patient management. Future prospective studies directly comparing PCCT with CBCT and conventional CT are essential to define task-specific clinical indications, optimize and standardize acquisition protocols, and evaluate radiation exposure and cost-effectiveness. With appropriate clinical validation, PCCT has the potential to become a valuable complementary imaging modality in oral and maxillofacial radiology, particularly in complex cases where high spatial resolution, spectral characterization, or metal-artifact reduction may provide additional diagnostic information.

## Figures and Tables

**Table 1:** Comparison of CBCT, EID-CT, and PCCT in Dental Imaging.

Parameter	CBCT	EID-CT	PCCT
Detector principle	Flat-panel X-ray detection [1]	Indirect conversion using scintillator-based detectors [2]	Direct photon-counting semiconductor detection [2]
Fine accessory canal delineation	In Rau et al., one CBCT system reliably depicted artificial canals down to 300 µm; 200-µm canals were not reliably depicted [4]	In Rau et al., the two EID-CT systems did not reliably depict 200- and 300-µm artificial canals [4]	In Rau et al., all artificial canals down to 200 µm were reliably depicted in the tested phantom [4]
Fine endodontic structures and root cracks	High-resolution 3D Accuitomo 170 performed similarly to PCCT for fine structures and better for root cracks in Fontenele et al. [5]	Not evaluated in the Fontenele et al. CBCT–PCCT comparison [5]	Similar performance to high-resolution 3D Accuitomo 170 for fine structures; inferior for root cracks in that study [5]
Spectral reconstruction	Conventional CBCT does not provide PCCT-type intrinsic spectral/VMI reconstructions [1]	Not assessed in the cited dental head-to-head studies [4,5]	Intrinsic spectral acquisition supports virtual monoenergetic imaging (VMI) [1,10]
Implant-related artifacts	Conventional dental imaging may be limited by metal-related artifacts [1]	Not evaluated in the cited peri-implant studies [7,10]	High-keV VMI reduced implant-related artifacts in an ex vivo titanium-implant study [10]
Peri-implant and bone visualization	Not directly compared with PCCT in Ruetters et al. [7]	Not directly evaluated in Ruetters et al. [7]	Detailed peri-implant, cortical and spongious bone depiction reported; quantitative trabecular microarchitecture not validated [7]
Effective dose: matched endodontic protocols	14.7/78.4/108 µSv (low/standard/high) in an ex vivo study [6]	Not evaluated in the matched-dose study [6]	13.1/75/102 µSv (low/standard/high) in the same study [6]
Effective dose: other endodontic study	0.03–0.13 mSv (standard-resolution) and 0.01–0.04 mSv (high-resolution) [5]	Not evaluated in this study [5]	0.85 mSv with the manufacturer-recommended head protocol [5]
Dose comparison with dental DVT	DVT: 4 mGy CTDI (16-cm phantom) in Sawall et al. [8]	Not evaluated in this study [8]	1 mGy CTDI (16-cm phantom) in Sawall et al.; one-quarter of DVT CTDI [8]

Note: Reported dose values are protocol- and task-specific and should be interpreted within the context of the individual studies.

**Table 2:** Methodological characteristics and principal findings of representative studies evaluating photon-counting CT in dental and maxillofacial imaging.

Study	Design and Sample Size	PCCT Scanner	Radiation Dose (PCCT Unless Noted)	Comparator	Principal Findings
[4] Rau (2025)	Phantom; 3 bovine teeth, 15 artificial canals (200–1000 µm)	NAEOTOM Alpha	CTDI 6.01 mGy; DLP 52.43 mGy·cm	2 CBCT; 2 EID-CT; micro-CT reference	All artificial canals ≥ 200 µm visualized; CBCT/EID-CT reliably depicted ≥300 µm.
[5] Fontenele (2023)	Anthropomorphic phantom (human skull and dentate mandible); 4 endodontic tasks	NAEOTOM Alpha	Estimated effective dose 0.85 mSv	3D Accuitomo 170 and NewTom VGi evo CBCT; micro-CT reference	Similar to high-resolution Accuitomo for fine endodontic anatomy; high-resolution CBCT better for root cracks.
[7] Ruetters (2025)	Ex vivo; 10 porcine mandibles, 30 titanium implants	NAEOTOM Alpha	CTDI (16-cm phantom) 4 mGy	Orthophos SL 3D CBCT; preimplant reference/physical bone measurements	Smaller mean bone-thickness deviation with PCCT (0.06 ± 0.08 vs. 0.39 ± 0.34 mm).
[8] Sawall (2024)	Ex vivo; 10 porcine jaws	NAEOTOM Alpha	CTDI (16-cm phantom) 1 mGy (PCCT) vs. 4 mGy (DVT)	Orthophos XL DVT	Higher dose-normalized contrast-to-noise ratio and subjective quality with PCCT at one-quarter DVT CTDI.
[10] Al-Haj Husain (2026)	Ex vivo; 6 porcine mandibles, 12 titanium implants	NAEOTOM Alpha	CTDIvol 3.28 mGy; DLP 45.9 mGy·cm; estimated effective dose 92 µSv	UHR, VMI 70–190 keV ± iMAR (within-PCCT comparison)	120–130 keV VMI minimized implant artifacts; iMAR did not improve quality.
[20] Klintström (2024)	Cadaveric; 3 sheep heads, 2 with inserted metal plates	NAEOTOM Alpha	CTDIvol (16-cm phantom) 3.83 mGy	EID-CT and intraoperative C-arm CBCT	Improved SNR and bone sharpness versus CBCT; no clear superiority over EID-CT.
[21] Sandhu (2026)	Ex vivo; 6 porcine mandibles, 33 osteosynthesis fixations with simulated fractures	NAEOTOM Alpha	PCCT effective dose 161 µSv (standard), 28 µSv (low)	CBCT at approximately matched dose levels	Better subjective fracture-line and fixation visualization, particularly at low dose.
[6] Al-Haj Husain (2026)	Ex vivo; 16 extracted third molars, 8 simulated endodontic tasks	NAEOTOM Alpha	PCCT effective dose 102/75/13.1 µSv (high/standard/low)	NewTom 7G CBCT, approximately dose matched	Reported diagnostic accuracy 80–88% vs. 75–88% (CBCT) for simulated tasks.
[19] Al-Haj Husain (2025)	Ex vivo pilot; 6 cadaveric pig mandibles, 36 induced fractures	NAEOTOM Alpha	PCCT median DLP 79.8/14.3 mGy·cm (standard/low)	NewTom 7G CBCT; physical ground truth	More accurate fracture-gap and hardware measurements, with improved reproducibility.
[9] Al-Haj Husain (2025)	Ex vivo; 6 porcine mandibles with simulated osseous lesions	NAEOTOM Alpha	PCCT estimated effective dose 360/122/20 µSv (high/standard/low)	Orthophos SL 3D CBCT	Higher subjective lesion detectability, especially at low dose; no patient-level validation.

Abbreviations: CBCT, cone-beam CT; CTDI, CT dose index; CTDIvol, volume CT dose index; DLP, dose–length product; DVT, digital volume tomography; EID-CT, energy-integrating detector CT; iMAR, iterative metal artifact reduction; PCCT, photon-counting CT; SNR, signal-to-noise ratio; UHR, ultra-high resolution; VMI, virtual monoenergetic imaging.

## Data Availability

No new data were created or analyzed in this study. Data sharing is not applicable to this article.

## Abbreviations

Abbreviation	Definition
OMFR	Oral and maxillofacial radiology
PCCT	Photon-counting computed tomography
CBCT	Cone-beam computed tomography
MDCT	Multidetector computed tomography
EID-CT	Energy-integrating detector computed tomography
VMI	Virtual monoenergetic imaging
iMAR	Iterative metal artifact reduction
DVT	Digital volume tomography
CNRD	Dose-normalized contrast-to-noise ratio
UHR	Ultra-high resolution
VSP	Virtual surgical planning
CAD/CAM	Computer-aided design/computer-aided manufacturing
TMJ	Temporomandibular joint
SPECT	Single-photon emission computed tomography
HNSCC	Head and neck squamous cell carcinoma
NIC	Normalized iodine concentration
ICC	Intraclass correlation coefficient
ALARA	As Low As Reasonably Achievable
ALADA	As Low As Diagnostically Acceptable
RME	Rapid maxillary expansion
FESS	Functional endoscopic sinus surgery
BMD	Bone mineral density
vBMD	Volumetric bone mineral density
DXA	Dual-energy X-ray absorptiometry
QCT	Quantitative computed tomography
HR-pQCT	High-resolution peripheral quantitative computed tomography
AI	Artificial intelligence
